# 2-Methyloxolane as a Bio-Based Solvent for Green Extraction of Aromas from Hops (*Humulus lupulus* L.)

**DOI:** 10.3390/molecules25071727

**Published:** 2020-04-09

**Authors:** Vincent Rapinel, Aziadé Chemat, Cyrille Santerre, Justine Belay, Farnaz Hanaei, Nadine Vallet, Laurence Jacques, Anne-Sylvie Fabiano-Tixier

**Affiliations:** 1 Green Extraction Team, Avignon University, INRAE, UMR408, F-84000 Avignon, France; vincent.rapinel@minakem.com (V.R.); aziade.chemat@alumni.univ-avignon.fr (A.C.); 2 Pennakem Europa, 224 avenue de la Dordogne, F-59944 Dunkerque, France; laurence.jacques@minakem.com; 3 Institut Supérieur International du Parfum, de la Cosmétique et de l’Aromatique alimentaire (ISIPCA), 34-36 rue du parc de Clagny, F-78000 Versailles, France; csanterre@isipca.fr (C.S.); jbelay@isipca.fr (J.B.); fhanaei@isipca.fr (F.H.); nvallet@isipca.fr (N.V.)

**Keywords:** 2-methyloxolane, hexane, green solvent, extraction, aromas

## Abstract

The potential of using the bio-based solvent 2-methyloxolane, also known as 2-methyltetrahydrofuran or 2-MeTHF, as an alternative to petroleum solvents such as hexane, was investigated for the extraction of volatile compounds from hop cones (*Humulus lupulus* L.). Lab scale extractions were coupled with in silico prediction of solutes solubility to assess the technical potential of this bio-based solvent. The predictive approach was performed using the simulation software COSMO-RS (conductor like screening model for real solvants) and showed that the 2-methyloxolane is as good as or better than hexane to solubilize the majority of aromas from hop cones. The experimental results indicated that the highest aroma yield was obtained with 2-methyloxolane with 20.2% while *n*-hexane was only able to extract 17.9%. The characterization of aromas extracted by the two solvents showed a similar composition, where lupulone was the main component followed by humulone. No selectivity of the solvents was observed for any of the major analytes. Finally, a sensory analysis was performed on the extracts, showing that both concretes using 2-methyloxolane and hexane have similar olfactory profiles. The results indicate that 2-methyloxolane could be a promising bio-based extraction solvent for hexane substitution.

## 1. Introduction

In solid–liquid extraction, it is commonly admitted that extraction efficiency strongly depends on the solvent, as described by Choi and Verpoorte [1]: “What you see is what you extract”. Conventional solvents, generally used in plant extraction, have the important advantage to present a good selectivity, relatively low boiling point, and low enthalpy of vaporization, which facilitates their removal after extraction and limits energy consumption. In particular, hexane is considered to be the best solvent for the extraction of lipophilic molecules such as lipids, aromas, and colors such as carotenoids [2] and the reason why it is widely used in the extraction industry. However, besides their obvious advantages, these solvents also present negative aspects. In most cases, they are obtained from non-renewable resources often petroleum-sourced, VOC (volatile organic compound) emitters, and harmful to human health and the environment. In particular, *n*-hexane, the major isomer of hexane, is known to be neurotoxic and hazardous for the environment [3,4].

Therefore, solvent selection is crucial in the demarche of green extraction so the following good practice guidelines [5] must be respected as much as possible to ensure the durability of the global process:

- Use of a natural origin, renewable, or agro-sourced solvent
- Avoid the use of solvents which might affect the safety and health of production operators and consumers. It must not be a CMR (carcinogenic, mutagenic, reproductively-toxic) or toxic (or present low toxicity), it must not induce an allergenic effect, and not belong to endocrine disruptors.
- Use of solvent compatible with existing in industrial facilities
- Prefer a solvent with a high rate of recyclability
- High bio-degradability and no bio-accumulation, to limit global process impact on the environment
- Use of solvent which limits energy consumption and cost of global process (solvent with low boiling point, low specific heat capacity, and low enthalpy of vaporization)
- Ensure a maximal solvent recovery at the end of the process using various available techniques

In the late 2000’s, a new solvent named 2-methyloxolane (CAS 96-47-9), also known as 2-methyltetrahydrofuran or 2-MeTHF, appeared in the solvents market. Originally used in the fine chemical industry [6,7] as an reaction solvent in substitution of THF (tetrahydrofuran), recent studies proved that 2-methyloxolane is a very promising “green” solvent for the extraction of bioactive components from natural sources. It is 100% produced from renewable biomass by hydrogenation of carbohydrate fractions, obtained by acid hydrolysis of hemicellulose from various feedstock [6,8,9]. It is biodegradable, has a promising environmental footprint, with a good preliminary toxicology assessment [10], and is easy to recycle. However, solvent substitution is a very long process that requires a lot of effort and research to convince the industry. Since 2012, several studies have been carried out to assess the potential of 2-methyloxolane as an alternative to petroleum solvent for extraction of oils [11,12,13,14], colors [15,16], and aromas [17,18].

In this context, this study aims to evaluate under the green chemistry concept the performance of 2-methyloxolane as a hexane substitution solvent for extraction of food aromas from hop cones. Hop was selected as the model of our study because it is a product used worldwide, particularly in the brewing industry to add bitterness and aroma to beer, with major components such as esters, aldehydes, ketones, terpenes, and carboxylic acids. A theoretical solubility analysis was initially carried out using a computational predictive method (conductor-like combination of quantum chemistry (COSMO)) coupled with statistical thermodynamics (real solvents, RS) to compare the solvation of targeted aromas metabolites in 2-methyloxolane and *n*-hexane. After silico approach and considering the physicochemical properties, we experimentally evaluate the ability of 2-methyloxolane compared with hexane to extract aroma concretes from hops cones. We performed appropriate comparisons in terms of solubility, aroma extraction yields, and economic vs. ecological parameters.

## 2. Results and Discussion

### 2.1. Solubility Study: COSMO-RS

**Figure 1:**
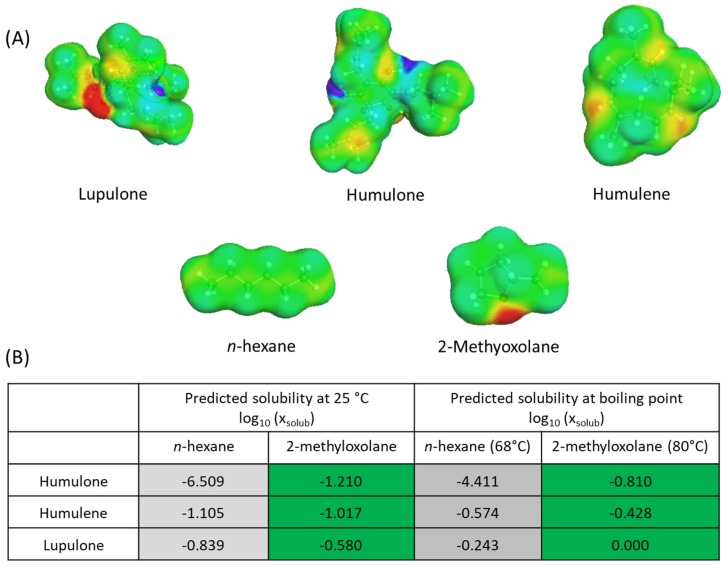
(**A**) Sigma surfaces of lupulone, humulone, humulene, *n*-hexane, and 2-methyloxolane. Yellow/red: molecule charge density < 0, blue: molecule charge density > 0, green: molecule charge density = 0. (**B**) Conductor like screening model for real solvents (COSMO-RS) results of solubility prediction of humulone, humulene, and lupulone in *n*-hexane and 2-methyloxolane at 25 °C and at boiling point. Grey: reference, green: better solvent than the reference.

Figure 1 shows COSMO-RS solubility predictions of some aromatic components in hops, such as humulone, humulene, and lupulone in *n*-hexane and 2-methyloxolane. These targeted solutes were chosen because they are known to represent the majority of aromatic compounds in hops. The simulations were carried out at 25 °C but also at the boiling temperatures of each solvent. The results, expressed as log_10_ (x_solub_), the logarithm of the molar fraction of solute in the solvent, show a much higher theoretical solubility of the analytes in 2-methyloxolane, in particular humulone and lupulone which are more polar than humulene. Indeed, both *n*-hexane and 2-methyloxolane have a large surface with a neutral charge density that allows favorable interactions with other lipophilic surfaces, in particular humulene, but the specificity of 2-methyloxolane comes from the presence of the oxygen atom in the oxolane group which also allows interactions with dipoles found in humulone (2 ketones and 3 hydroxyl groups) and lupulone (2 ketones and 2 hydroxyl groups) (see Figure 1A). Therefore, COSMO-RS calculations (see Figure 1B) predicted much higher solubilities of oxygenated compounds, such as humulone (×10^5^ times more at 25 °C) and lupulone, but also an almost equal solubility for humulene, an apolar compound. As a reminder, *n*-hexane is one of the reference solvents for solubilization of lipophilic compounds. It must be also noted that, as expected, predicted solubilities increased with the temperature, more or less depending on the solute/solvent.

In conclusion, COSMO-RS calculations predicted that 2-methyloxolane offers a better solubilization than *n*-hexane for the major aromatic compounds that are usually found in the hop concretes. This is particularly visible in the case of more polar compounds such as humulone or lupulone. This first study in silico represents an important first step in determining whether 2-methyloxolane can successfully substitute hexane for the extraction of hop flavors.

### 2.2. Extraction Yields

The extraction yields obtained for both solvents using maceration and Soxhlet extraction are shown in Table 1. As mentioned in the literature, extractions using 2-methyloxolane often gave higher extraction yields compared to hexane, regardless of the extraction technique used. Here, 2 h macerations under reflux also resulted in higher crude extraction yields using 2-methyloxolane (16.6%) in comparison with hexane (12.7%). Total crude extraction yield, evaluated using Soxhlet extraction (6 h), also resulted in higher yield with 2-methyloxolane (20.2% vs. 17.9% with hexane). The difference of extraction yield can be explained by several factors, including (i) the higher extraction temperature due to the higher boiling point (80 °C vs. 68 °C); (ii) the higher solvation power; (iii) a larger extraction spectrum, from apolar to slightly polar molecules, as predicted by COSMO-RS; (iv) presence of residual solvent trapped in the waxy extract.

Interestingly, it must also be noted that the extraction kinetic seems faster in the case of 2-methyloxolane, as a 2 h maceration extracted 82% of the total crude yield whereas hexane only extracted 71%. These results could obviously be linked to the higher extraction temperature (extraction under reflux) but some authors also found similar results for extractions, both under reflux [2] and at the same temperature (55 °C) [12]. This information is crucial for industrial process equipment dimensioning, as it directly influences the residence time in the extractor, and thus the productivity.

### 2.3. Chemical Compositions

Hop aromas are a complex mixture of volatile substances including monoterpenes, sesquiterpenes, and volatile alcohols, as well as prenylated-phloroglucinol derivatives known as “α-acids”, responsible for the bitterness of hops, and “β-acids”. The constituents in *Humulus lupulus* (hops) essential oil are mainly β-myrcene, linalool, α-humulene, and β-caryophyllene. However composition is not consistent across years or cultivars [19]. The α-acids, particularly humulone, cohumulone, and adhumulone are important constituents determining the quality of hops [20]. β-acids, mainly lupulone, are often regarded as undesirable as they are prone to auto-oxidation and often cause off-flavor problems [21]. A previous study carried out by Filly et al. [17] showed that 2-methyloxolane could be a credible bio-based and non-toxic alternative to hexane for extraction of blackcurrant buds’ aromas. Here, 2-methyloxolane was evaluated as an alternative to hexane for the extraction of aromas from hop pellets using solid–liquid extraction. Only maceration extracts were analyzed in this study as Soxhlet extracts were not considered representative of what could be obtained in the industry. Composition of the essential oil was also determined as a reference for the olfactive part.

In the context of hops aromas extraction, we analyzed the extracts by gas chromatography–mass spectrometry (GC-MS) and flame ionization detector (FID) to investigate if the higher extraction yields and the difference in terms of solubility could also lead to differences in the chemical composition of the extracts (see chromatograms in Figure S1). Compositions of both solvent extracts were compared with essential oil using GC-MS/FID. In total, 87 compounds were detected (Table 2), with respectively 66 compounds in the essential oil, 19 in the hexane maceration extract, and 15 in the 2-methyloxolane maceration extract.

Finally, both solvents showed a relatively similar chemical profile with a majority of bitter acids and derivatives (>70%), in particular α-acids (17.9% and 19.1%, respectively). The most significant variation between both solvents is the proportion of lupulone (β-acid), lower in the hexane extract (41% vs. 60%). On the contrary, humulene contents are very close (1.42 vs. 1.16%). This result is in agreement with the solubility predictions made using COSMO-RS.

However, some deviations from the predictions must be noted. First, hexane extract has quite a high amount of hulupone (9.3%), known to be a oxidation product of lupulone [21,22]. As this compound was not detected in the 2-methyloxolane extract and because there is no reason that hulupone is selectively extracted by hexane, it was assumed that (i) it was due to an overheating or air exposure during extraction or desolvantization steps; (ii) as hulupone is very photosensitive [23], it was degraded by UV light in the 2-methyloxolane extract due to prolongated storage in the light. Then, no major differences were observed for humulone and isohumulone content, even though they should be theoretically more soluble in 2-methyloxolane due to their more polar nature.

In conclusion, COSMO-RS is a helpful tool for the solvent selection process, however the solubility values alone cannot represent the complexity of the extraction processes, in particular the intra-particle diffusion phenomenon, and should not be seen as a tool able to predict the quantitative composition of an extract. In addition, it must be noted that desolventization, in particular for this kind of “pasty” material, and storage can have a major impact on the extract composition.

As expected, essential oil and solvent extracts have very different compositions. Hops essential oil is mainly composed of monoterpenes and sesquiterpenes, in particular β-Myrcene (35%), α-Humulene (22%), E-Caryophyllene (9%), and E-β-Farnesene (7%), but shows no traces of bitter acids, and not enough volatiles to be stripped.

### 2.4. Sensory Analysis

**Figure 2:**
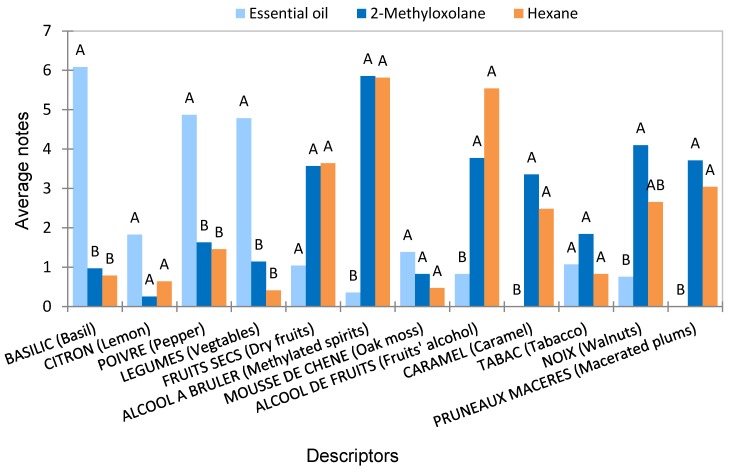
Olfactory descriptors used by the sensory panel to evaluate the essential oil and the two hop extracts. ANOVA with a threshold of 5% followed by Fisher’s test to determine the significant differences between the averages notes of the three samples. Products with the same letter are not significantly different.

Results of the sensory analysis (Figure 2) showed that, as expected, the olfactory profile of the essential oil is significantly different from the concretes, showing very intense “basil”, “pepper”, and “vegetables” olfactory notes, significantly less intense for “Fruit alcohol” and “methylated spirit” and an absence of “macerated plums” and “caramel” notes. In opposite, both hop extracts showed similar olfactory profiles independent of the solvent type. The predominance of the descriptors “alcohol” and “methylated spirit” in the solvent extracts in comparison with the essential oil is in accordance with their chemical composition (<30% of odorous molecules). “Walnuts”, “tobacco”, and “caramel” flavors that were also used to describe solvent extracts could be related to the presence of oxidation products generated during desolventization. In conclusion, the two hop extracts gave very similar olfactory profiles except for a slightly more intense “walnuts” note for the 2-methyloxolane extract.

### 2.5. Techno-Economic Comparison

In addition to the extraction capability assessment and extracts quality, we evaluated 2-methyloxolane and *n*-hexane under technical, ecological, and economical aspects. In particular, Table 3 shows that 2-methyloxolane as a green solvent is produced 100% by biochemical transformations of agro-building blocks (i.e., defined molecules obtained from the biomass feedstock) [2]. In contrast hexane is currently obtained 100% from petroleum and is known to be hazardous for the environment and health. Regarding toxicity, recent results comparing non-observed adverse effect concentrations (NOAEC), used to determine the maximal safe concentration of a compound in the air for workers, showed that hexane’s NOAEC is 23 times lower than 2-methyloxolane (122 ppm vs. 2838 ppm) [24,25]. Regarding environmental impact, a recent study [26] showed that the CO_2_ emissions to manufacture 1 kg of solvent were 10 times lower for 2-methyloxolane than for conventional solvent such as hexane (0.19 vs. 1.9 kg CO_2_/kg). Finally, a previous study by Sicaire et al. on rapeseed oil extraction [12] showed that even if the solvent price is higher than hexane (8–9 €/kg vs. 1 €/kg [24]) and the energy necessary to vaporize 1 kg of solvent (vaporization enthalpy) is 10% higher for 2-methyloxolane, it can be compensated at the industrial scale thanks to the higher extraction yield and faster extraction. For more valuable extracts such as aromas and perfumes, the extra-cost due to the solvent can be even more easily compensated, without considering any “premium” price that could be applied due to the “greener” aspect of the extracts.

## 3. Materials and Methods

### 3.1. Plant Material and Chemicals

**Figure 3:**
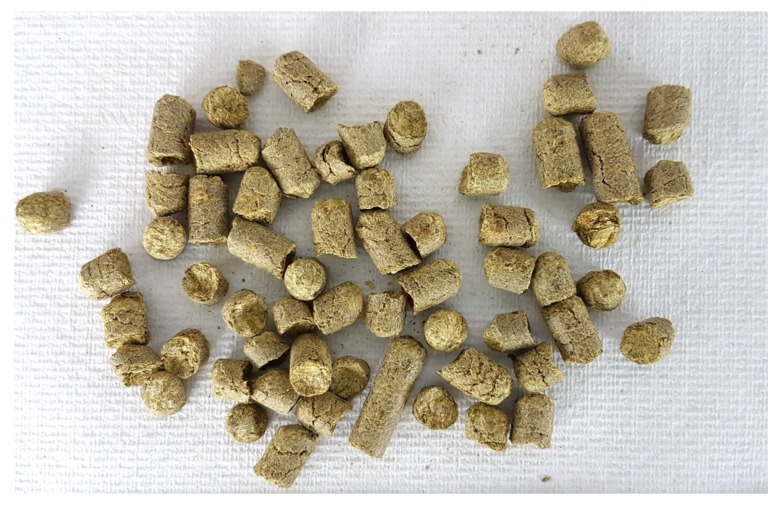
Hop cones pellets (diameter = 6 mm; length = 5–20 mm).

Hop cones (*Humulus lupulus* L.; variety “Cascade”; harvest 2017; α = 6.5, Figure 3) were purchased as pellets from a local supplier (France). For chemicals, both 2-methyloxolane (peroxides inhibitor:BHT) and hexane (technical grade) used for extractions were purchased from VWR International (Radnor, PA, USA). Solvents and reagents used for analysis were analytical grade.

### 3.2. Computational Method: COSMO-RS Calculations

The conductor-like screening model for real solvents (COSMO-RS) was used to predict and compare the solubility of some selected solutes into 2-methyloxolane and *n*-hexane.

Briefly, COSMO-RS is a calculation method developed by Klamt [28] using a quantum chemistry model based on the prediction of chemical potential of a substance in the liquid phase. COSMO-RS can be used as powerful tool for extraction solvent screening [29,30,31,32,33].

COSMO-RS procedure comprised two steps at different scales: a microscopic scale step followed by a macroscopic scale step. First, the COSMO model is used to apply a virtual conductor environment for the molecule, inducing a polarization charge density on its surface: the σ-surface (Figure 1A). The green color corresponds to a charge density equal to 0, the blue color to a positive charge density (δ^+^), and the red color to a negative charge density (δ^−^). The molecule structure and the charge distribution are then optimized in order to obtain the minimal energy of the system using algorithm-based calculations (density functional theory).

Based on the obtained polarization charge density, the solute interaction energy is quantified using a statistical thermodynamic calculation. The spatial distribution of the polarization charge is converted into a composition function: σ-profile. This σ-profile provides information about the molecular polarity distribution. At this stage, the molecule is considered isolated so interactions with neighboring molecules are not taken into account. In order to consider the molecule as a solvent (or a solute in a solvent), this function is then integrated to calculate the chemical potential of the surface (σ-potential) using COSMOthermX program (version C30 release 16.02). The σ-potential can be interpreted as the affinity between a solvent S and the surface σ. Solvent interactions are reduced to a combination of local interactions between a pair of surface portions with charge densities σ and σ’. The interaction energy functional E_int_ is defined as the sum of three contributions (Equation (1)).

E_int_ = E_misfit_ + E_hb_ + E_vdw_ (1)

where E_misfit_ is the interaction energy between two surfaces of different charge density; E_hb_ is the hydrogen bonding energy; E_VdW_ is Van der Waals interactions.

Statistical thermodynamic calculations are then used to calculate macroscopic properties from these molecular interactions. In particular, the determination of the chemical potential is used to predict almost all thermodynamic properties of compounds or mixtures of compounds, including solubility. In practice, the software COSMOthermX calculates the theoretical solubility of a solute in the solvent, expressed as the log of the mole fraction of the solute: log_10_ (x_solub_). The relative solubility is calculated from the following equation (Equation (2)):

(2) $$\log_{10}\left(x_{i}\right)=\log_{10}\lfloor \frac{\exp({µ}_{i}^{pure}-{µ}_{i}^{solvent}-\Delta G_{i,fusion}}{RT}\rfloor$$

where μ*_i_^pure^* is the chemical potential of pure compound *i*; μ*_i_^solvent^* is the chemical potential of *i* at infinite dilution; Δ*G_i,fusion_* is thte free energy of fusion of *i*; *x_i_* is molar solubility of *i*.

Relative solubility is always calculated in infinite dilution. The log_10_ (x_solub_) of the best solubility is set to 0 and all other solvents are given relatively to the best solvent. As an example, a solute with log_10_ (x_solub_) = −1 in a solvent S has, in theory, a solubility which is 10 times lower than the same solute in the best solvent (log_10_ (x_solub_) = 0). Calculations were performed at 25 °C and at boiling point (68 °C for hexane, 80 °C for 2-methyloxolane), using the “iterative” mode, considering that solutes and solvents are pure and in a liquid state. Reliability of the COSMO-RS simulations in the context of natural product extraction was assessed by Filly et al. [29].

### 3.3. Extraction Yield

Crude extractions were calculated using the following equation (Equation (3)):

(3) $$\mathrm{Extraction}\;\mathrm{yield}\;\left(\%\right)=\frac{\mathrm{mass}\;\mathrm{of}\;\mathrm{dry}\;\mathrm{extract}\;\left(g\right)}{\mathrm{mass}\;\mathrm{of}\;\mathrm{starting}\;\mathrm{material}\;\left(\mathrm{dry}\;\mathrm{basis}\right)\;\left(g\right)}$$

### 3.4. Solid–Liquid Extraction

Briefly, 17 g of hop pellets were soaked in 175 mL of solvent (hexane or 2-methyloxolane) and then heated under reflux for 2 h. Extracts were filtered under reduced pressure through some cotton wool, then the solvent was evaporated using a rotary vacuum evaporator (40 °C; 130 mbar; 30 min). As the dry extracts obtained were very pasty, they were also desolventized using a vane-cell pump with heating (≈60 °C) for 15 min. Resulting dry extracts were weighed to calculate the crude extraction yield and finally stored at −20 °C before analysis. Each extraction was made in triplicate.

### 3.5. Soxhlet Extraction

In order to calculate the maximal extraction yield for both solvents, hop pellets were extracted using a Soxhlet apparatus using the following conditions: about 30 g of hop pellets were introduced in cellulose cartridges and placed in the extraction chambers (200 mL). About 200 mL of solvent were introduced in the bottom solvent flask and heated so the duration of the cycle was around 6.5 +/− 0.5 min. The total duration was set at 6 h. The resulting extracts were desolventized using the same protocol previously described.

### 3.6. Production of Essential Oil

Hop essential oils were also produced to get a comparison basis for chemical composition and sensory analysis, using the following procedure: 250 g of hop pellets were soaked in 2.5 L of water and hydro-distillated using a Clevenger-type apparatus for 4 h (3500 W). The essential oil was then collected and dried under anhydrous sodium sulfate. Extraction was made in duplicate.

### 3.7. Identification of Aroma by GC-MS

The chemical composition of each extract was determined by GC-MS analysis for the characterization and GC-FID to estimate relative quantities of each analyte. Samples were diluted at 10% in ethanol. Liquid injection was conducted with an autosampler installed on an Agilent 7890GC. The injection volume was set as 1.6 µL using a split ratio of 100:1. The following parameters were used for GC-MS analyses: injector temperature 250 °C, column SLB^®^-5ms (30 m × 0.25 mm × 0.25 mm); temperature, from 40 °C to 300 °C at 5 °C/min and maintained for 5 min; carrier gas, helium; flow rate, 1 mL/min. A 5977 simple quadrupole mass spectrometer (Agilent Technologies, Santa Clara, CA, USA) was used for identification. Mass spectra were recorded in electron ionization mode at 70 eV. The transfer line and the ion source were set at 250 °C. Mass spectra were scanned in the range *m*/*z* 30–400 Da. Compound identification was carried out by comparison of mass spectra recorded and mass spectra from several libraries (Adams, Institut Supérieur International du Parfum, de la Cosmétique et de l’Aromatique alimentaire (ISIPCA), and NIST—National Institute of Standards and Technology). For pseudo-quantification a splitter was used after the column and before FID (1/3 of the signal was sent to MS and 2/3 to FID).

Each analysis was performed in triplicate and tridecane was added at 0.1% as the internal standard. Proportions of each analyte found in the volatile fraction are given in Table 2, after subtraction of the solvent (hexane or 2-methyloxolane) and peroxides inhibitor (BHT) signals.

### 3.8. Sensory Analysis

Sensory profiling was used to discriminate the main aromatic characteristics of hop extracts. The ISIPCA sensory olfaction panel, composed of eight highly trained women (45–70 years), participated in this study. Attribute generation was performed during six training sessions. After the elimination of non-relevant descriptors and the selection of the most representative terms, the final vocabulary consisted of twelve attributes (Figure 2).

Three samples were analyzed: hop essential oil, hexane concrete, and 2-methyloxolane concrete. Extracts were 10% diluted in ethanol, filtered, and then stored at 4 °C before the test. Scent cards were dipped 2 cm in each extract for 2 s and were then placed in closed bottles to generate a head space 2 h before evaluation. The samples were presented in a monadic way and were distributed using a Williams latin square design for each subject. To evaluate the intensity of each olfactory descriptor, the panel used a linear scale from 0 (no smell) to 10 (very intense smell). Results of the sensory evaluation are expressed as mean intensity value for each descriptor and for each extract. Analysis of variance (ANOVA) was performed on the sensory descriptive data to reveal significant sensory differences between samples.

## 4. Conclusions

The aim of this study was to evaluate the potential of 2-methyloxolane as a green, non-toxic, and biodegradable solvent to substitute hexane for the extraction of aromas from hope cones. A multi-factors approach was used, including solubility prediction using the COSMO-RS model, lab-scale extractions, GC-MS analysis of the extracts, sensory analysis, and techno-economical study.

COSMO-RS simulations showed that the major molecules responsible for the hop aromas are more soluble in 2-methyloxolane than in *n*-hexane, in particular oxygenated compounds. However, solubility predictions were not always in accordance with the chemical compositions determined by GC-MS. Solvent extracts were mainly composed of α- and β-acids (mainly lupulone and humulone) but with some differences. In particular, 2-methyloxolane extract has a higher proportion of lupulone than hexane (60% vs. 41%) but lower degradation products, in particular, hulupone found in the hexane extract (9.3%). Sensory evaluation of the two hop extracts by an olfactory expert panel resulted in the same olfactory profiles with one slight difference (more intense “Walnuts” note). Still, as extraction brings few modifications in terms of olfactory perceptions, the solvent substitution for aromas and perfume producers will probably not be immediate and should require the creation of a new product.

Quantitatively, extractions using 2-methyloxolane resulted in higher extraction yields (20.2% vs. 16.6% for hexane) with a faster kinetic (17.9% yield after a 2 h maceration vs. 12.7% with hexane). These differences are likely to be caused by the solvent itself, the higher extraction temperature, and the larger extraction spectra.

In addition to those results, we also showed that 2-methyloxolane has very interesting properties in adequation with the concepts of green extraction (bio-based, low toxicity, low CO_2_ emissions, etc.). In addition, 2-methyloxolane can be readily transposed to existing extraction units using hexane without major modifications and without excessive over-cost despite its higher cost. Therefore, we conclude that 2-methyloxolane is a very promising green solvent to replace hexane for the production of green aromas and is also an opportunity for the industry to create clean and innovative products.

## Figures and Tables

**Table 1:** Extraction Yields.

Technic/Solvent	Hexane	2-Methyloxolane
Maceration (2 h)	12.7% +/− 0.7	16.6% +/− 0.5
Soxhlet (6 h)	17.9% +/− 0.2	20.2% +/− 0.3

**Table 2:** Relative abundances of analytes (>1%) found in hops maceration extracts.

Compound	CAS	RI	EO	Hexane	2-Methyloxolane
Methacrolein	78-85-3	568	n.d.	0.63	1.25
2-methyl-3-Buten-2-ol	115-18-4	606	n.d.	5.65	2.30
Diethyl acetal	105-57-7	717	n.d.	5.32	1.91
β-Myrcene	123-35-3	991	35.03	n.d.	n.d.
Linalool	78-70-6	1100	1.20	n.d.	n.d.
2,3,4-Trimethyl-2-pentanol	66576-26-9	/	n.d.	2.55	1.45
E-Caryophyllene	87-44-5	1444	8.87	n.d.	n.d.
E-β-Farnesene	18794-84-8	1458	6.96	n.d.	n.d.
α-Humulene	6753-98-6	1467	22.34	1.42	1.16
γ-Murolene	30021-74-0	1475	1.28	n.d.	n.d.
β-Selinene	17066-67-0	1485	1.51	0.66	0.44
Geranyl isobutanoate	2345-26-8	1515	1.55	n.d.	n.d.
α-Selinene	473-13-2	1494	1.56	n.d.	n.d.
γ-Cadinene	39029-41-9	1512	1.05	n.d.	n.d.
δ-cadinene	483-76-1	1530	1.38	n.d.	n.d.
Caryophyllene oxide	1139-30-6	1573	1.61	2.40	2.07
Humulene oxide II	19888-34-7	1642	2.72	1.79	2.20
*E*,*Z*-1,3-Cyclododecadiene	1129-92-6	/	1.56	n.d.	n.d.
Phytol	150-86-7	/	n.d.	2.00	2.17
Hulupone	468-62-2	/	n.d.	9.30	n.d.
Isohumulone	25522-96-7	2715	n.d.	6.00	8.93
R-Humulone	26472-41-3	2740	n.d.	11.89	10.19
Lupulone	468-28-0	/	n.d.	41.34	60.22
Limonin	1180-71-8	/	n.d.	4.52	4.15

RI: Kovats retention index; EO: essential oil; n.d.: non-detected.

**Table 3:** Technical and ecological parameters of solvents used experimentally.

Solvent	Bp (°C) [27]	Log P [27]	Vaporization Enthalpy (kJ/kg) [7]	Safety Symbols	Resource	CO_2_ Footprint (kg/kg) [26]
***n*-hexane**	68	4.00	334	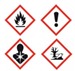	100% Petroleum	1.9
**2-methyloxolane**	80	1.85	364	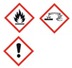	100% Cereal crop	0.19

## Supplementary Materials

The following are available online, Figure S1: GC-MS chromatograms of the essential oil, hexane maceration and 2-methyloxolane maceration extracts.
