# Supplementary material for: 2-Methyloxolane as a Bio-Based Solvent for Green Extraction of Aromas from Hops (Humulus lupulus L.)
Source: Molecules. 2020 Apr 9;25(7):1727. doi: 10.3390/molecules25071727 (PMC7180635; doi:10.3390/molecules25071727)
Supplement: Supplementary file 1 [file molecules-25-01727-s001.pdf]

molecules

MDPI

Supplementary materials

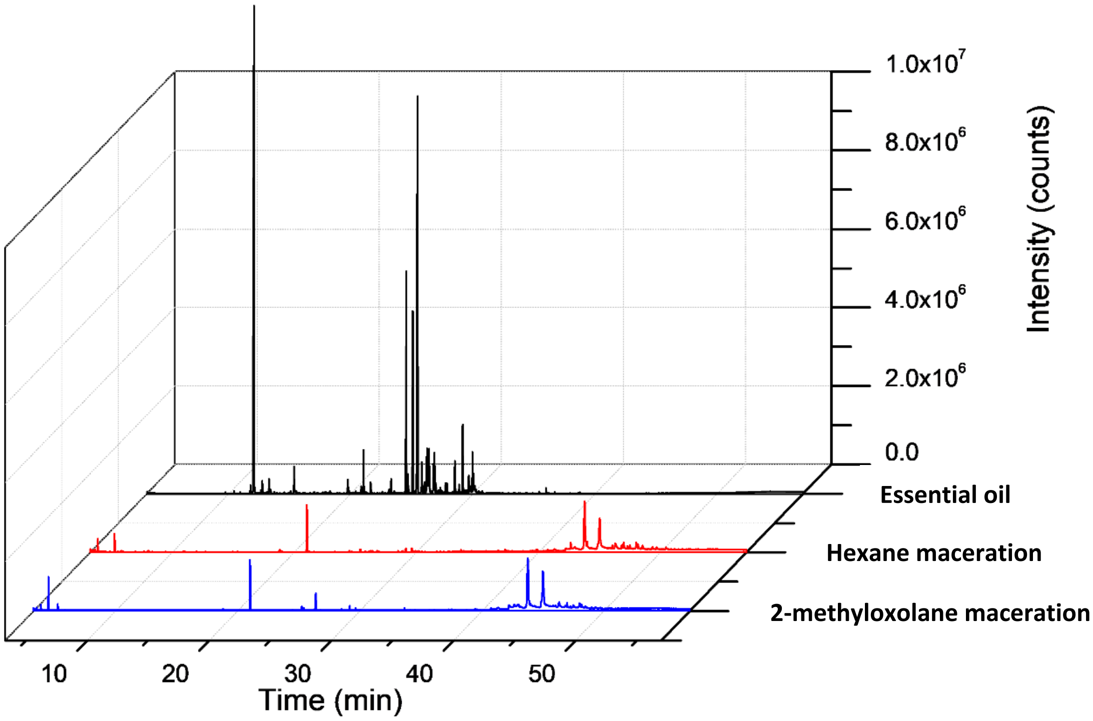

Figure S1 – GC-MS chromatograms of essential oil (top), hexane maceration (middle) and 2-methyloxolane (bottom) maceration extracts.
